# Antihypercholesterolemic Effects of Fruit Aqueous Extract of* Copernicia prunifera* (Miller) H. E. Moore in Mice Diet-Induced Hypercholesterolemia

**DOI:** 10.1155/2017/6376173

**Published:** 2017-06-11

**Authors:** Raquel Teixeira Terceiro Paim, Stephen Rathinaraj Benjamin, Davide Rondina, Márcia Maria Mendes Marques, Daniel de Araújo Viana, Maria Leônia da Costa Gonzaga, Ícaro Gusmão Pinto Vieira, Francisca Noélia Pereira Mendes, Paula Alves Salmito Rodrigues, Maria Izabel Florindo Guedes

**Affiliations:** ^1^Northeast Biotechnology Network, Graduate Program of Biotechnology, State University of Ceará, Itaperi Campus, 60714-903 Fortaleza, CE, Brazil; ^2^Laboratory of Biotechnology and Molecular Biology and Health Science Center, State University of Ceará, Itaperi Campus, 60714-903 Fortaleza, CE, Brazil; ^3^Faculty of Veterinary, State University of Ceará, Itaperi Campus, 60714-903 Fortaleza, CE, Brazil; ^4^Federal University of Piauí, Campus Senador Helvídio Nunes de Barros, Junco, 64607-670 Picos, PI, Brazil; ^5^Laboratory of Veterinary Pathology, State University of Ceará, Itaperi Campus, 60740-000 Fortaleza, CE, Brazil; ^6^Federal University of Ceará, University Campus of Pici, 60356-000 Fortaleza, CE, Brazil; ^7^Laboratory of Natural Products, State University of Ceará, Itaperi Campus, 60740-000 Fortaleza, CE, Brazil

## Abstract

The present objective of the investigation is to evaluate the antihypercholesterolemic activity of the aqueous fruit pulp extract (APE) of* Copernicia prunifera* (Miller) H. E. Moore (Arecaceae family). Various chemical characterization methods like thin layer chromatography, Fourier transform infrared spectroscopy, ^1^H and ^13^C NMR, and molecular weight by gel permeation chromatography have been employed to characterize the extracted pectin. The present study demonstrated that hypercholesterolemic diet (HD) created hypercholesterolemia, caused significant increases in body weight, total cholesterol, and low-density lipoprotein, and caused decreases in high-density lipoprotein in serum compared with SD group. Two doses (APE 150 and 300 mg/Kg b.w./day) were administered to hyperlipidemic mice for 90 days. APE reversed body weight changes, changed serum lipids to normal values, and significantly inhibited the changes of lipid peroxidation and inflammation in the liver tissues. The renal parameters analyzed (urea and creatinine) altered by diet were reverted to normal values. Our results revealed that aqueous fruit pulp extracts of carnauba reduced hypercholesterolemia showing a potential preventive effect against cardiovascular diseases without side effects cause.

## 1. Introduction

Atherosclerosis has among its main determinants hypercholesterolemia characterized by increase of low-density lipoprotein cholesterol (LDL-C) and triglycerides (TG) serum. This condition raises the important risk factors for cardiovascular disease (CVD) in the leading cause of mortality worldwide [1]. Cardiovascular disease, recently, in different geographical regions of the world is more prevalent in western countries and people with older age, thus giving a high incidence of mortality [2].

Over the last decades, in Brazil, 32% of deaths in adults represented the highest rate of death from cardiovascular disease in the Latin American region [3]. There are numerous factors that contribute to the abnormality of plasma lipids among which are modern lifestyle, high cholesterol, diet, and inadequate physical activity [4].

Many medications have been used to manage atherosclerosis over the years. These drugs on the market are very effective against deleterious effects of dyslipidemia acting mainly on the reduction of LDL-C and plasma triglycerides. Nevertheless, with prolonged use of these medications various side effects may arise such as liver and muscle toxicity, rhabdomyolysis, myopathy, and acute renal failure that may interrupt treatment. Therefore, the research of natural products with nutraceutical effects gained much prominence in several chronic diseases such as hypertension, hypercholesterolemia, and cancer [5].

Nowadays, nutraceuticals have received considerable interest due to potential nutritional, safety, and therapeutic effects. Among the many products, pectin stands out for being considered a dietary fiber that can be used in capsule or concentrated food formulations [6].

Pectins are complex polysaccharide constituents which consist of *α*-1,4-linked D-galacturonic acid, which is partly methyl esterified, and the side chain contains various neutral sugars (mainly L-rhamnose, L-arabinose, and D-galactose) [7]. However, other polysaccharides consisting of arabinose and/or galactose have been isolated with association with pectic polysaccharides such as arabinogalactan (AG) (type I and type II). The AG-II may be associated with proteins, called arabino galactan-proteins (AGPs) [8].

Carnauba palm or Brazilian tree of life (*Copernicia prunifera *(Miller) H. E. Moore, Arecaceae family) is a palm tree found in the northeastern Brazil and cerrado biomes, located in some Brazilian regions. Rufino et al. [9] evaluated the quality for fresh consumption and processing some nontraditional, tropical fruits in Brazil revealed that the carnauba's fruits had one of the highest pectin contents compared to the studied fruits (1.49%) like açai (0.96%), bacuri (0.56%), cashew (0.15%), and mangaba (0.48%).

Carnauba fruit is commonly used for animal feed [10, 11]. Besides that, carnauba leaves are noted for their elevated content of wax which has many applications in the pharmaceutical industry, food industry, cosmetics, and lubricants [12].

In recent years, numerous bioactive compounds in fruits have been reported for their antihypocholesterolemic effects in animals, especially pectin from red ginseng [13], orange, and apple [14] and penta-oligosaccharide pectin extracted from* Crataegus pinnatifida* Bunge Var.* major* [15]. The results revealed great application prospects of pectin in the development of functional food and nutracetuticals for the prevention of hyperlipidemia and dyslipidemia diseases. The diester of the 4-methoxycinnamic acid extracted from leaves of carnauba reduced cholesterol and plasma triglycerides dyslipidemic when tested in animals induced by diet [16].

The hypercholesterolemic effect of carnauba fruits has not yet been explored. In the current study, potential effects of pectin from unripe carnauba fruit aqueous pulp extracts were further investigated for its hypocholesterolemic activity in experimental animals induced by hypercholesterolemic diet.

## 2. Materials and Methods

### 2.1. Plant Materials

The unripe fruits of* Copernicia prunifera* (Mill.) H. E. Moore were collected in the Aracati, Ceará (northeast Brazil), and used for extraction. The aqueous fruit pulp extract (APE) was prepared by extracting the unripe fruits (100 g) which were subjected to delipidation with hexane followed by methanol and water. Then, the extract was filtered and concentrated. The concentrate was lyophilized in a freeze-dryer and stored at −20°C until use and the yield was approximately 2.15% further being evaluated by in vivo study. Other chemicals were purchased from Sigma (St. Louis, MO).

### 2.2. Isolation and Purification of Pectin from the* C. prunifera*

The pulps were separated from 100 g of unripe fruit from the* C. prunifera*. Moore was used for extraction of pectin by applying a 0.25% ammonium oxalate solution (pH 4.6) for 1 hour at 80°C, followed by a solid: liquid extraction at a ratio of 20 : 1. The extract was filtered after the extraction. The filtered extracts were combined, and the pH was adjusted to 6 with a 0.1 N sodium hydroxide solution; the extracts were then concentrated to a volume of 100 mL (10 : 1). Then, 300 mL of 95% ethanol was added to the concentrate to precipitate the pectin. The pectin was then filtered with a Buchner funnel. The filtration residue was dissolved in 100 mL of distilled water and filtered through a layer of celite. This operation was repeated until obtaining a transparent liquid that was then lyophilized to get the yield 2.3% further being characterized by spectroscopic and chromatographic methods.

### 2.3. Hydrolysis of Pectin

The hydrolysis is carried out according to the methodology of Kapoor and Joshi [17]. 200 mg sample of pectin was subjected to treatment with 10 ml of sulfuric acid solution 20% (v/v) for 3 hours. Then the mixture was neutralized with barium carbonate, filtered, and concentrated under vacuum and a temperature of 50°C to obtain a solid residue, which was dissolved in 10 ml of distilled water.

### 2.4. Thin Layer Chromatography (TLC)

TLC was performed on the 20 × 7 cm plates precoated with microcrystalline cellulose (Camag, Muttanez, Switzerland) using the cromatoplaca TLC silica gel 60 F_254_ plates. A volume of 1 *µ*L of 1% methanolic solutions of standards and investigated extracts was spotted on the plates. One-dimensional TLC analysis was performed with glacial acetic acid, chloroform, and distilled water in volume ratio 98 : 86 : 16 v/v as mobile phase. Spots were observed under UV light at 366 nm before and after spraying with orcinol-sulfuric acid solution.

### 2.5. Fourier Transform Infrared (FTIR) Analysis

Analysis were performed on a Shimadzu 8300 FTIR spectrophotometer to investigate the characteristic spectra of the extracted pectins. Dried sample (1 mg) and potassium bromide (100 mg) were mixed, ground, and pressed into tablets. The spectra were scanned within the range of 4000–400 cm^−1^.

### 2.6. ^1^H, ^13^C Nuclear Magnetic Resonance (NMR)

^1^H and ^13^C NMR spectra experiments were carried out in a Bruker Avance DRX-500 spectrometer running at 500 MHz (^1^H) and 125 MHz (^13^C), at room temperature, with the acquisition parameters (45° pulse width and 1.0 s relaxation delay). In ^1^H (300 MHz) spectra the chemical shifts (expressed in ppm) were referenced to the internal standards: TMS (tetramethylsilane) and DSS (2,2-dimethyl-2-silapentane-5-sulfonate). ^13^C (75 MHz) spectra with CDCl_3_ (deuterochloroform) and CH_3_OD (methanol deuterated), respectively, were referenced to standard absorptions at *δ* 77.0 and *δ* 49.0, respectively, and with D_2_O (deuterated water) to DSS, at a ^1^H frequency of 500 MHz and an expansion of ^1^H NMR.

### 2.7. Gel Permeation Chromatography (GPC)

The peak molar mass (*M*_pk_) of pectin was determined by gel permeation chromatography using a Shimadzu instrument (ultrahydrogel linear column, 7.8 × 300 mm), at room temperature, flow rate of 0.5 ml/min, polysaccharide concentration of 0.1% (w/v), and 0.1 M NaNO_3_ (sodium nitrate) as the solvents. A differential refractometer was used as the detector. The elution volume was corrected by the use of the internal marker ethylene glycol at 11.25 ml. Pullulan samples (Shodex Denko) of molar mass 5.9 × 10^3^, 1.18 × 10^4^, 2.12 × 10^5^, and 7.88 × 10^5^ g/mol were used as standards.

### 2.8. Animals and Experimental Design

The antihypercholesterolemic activity of the extract was carried out on 35 male Swiss mice aged 6–8 weeks and weighing 25–30 g prior to the experiment. All animals had free access to standard diet food and tap water ad libitum. Animals were kept at 22 ± 2°C under a 12 h/12 h light dark cycle. Food intake and body weights were recorded once a week throughout the experiment. Animals were housed in standard environmental conditions in the Laboratory of Biotechnology and Molecular Biology of the State University of Ceará (Fortaleza, Ceará). All animal experimental procedures were approved by the Ethics Committee in Animal Experimentation of Ceará State University (Comissão de Ética para o uso de animais da Universidade Estadual do Ceará, CEUA) under number 4558299/2016. The mice were randomly divided into 5 groups (*n* = 7): group 1: standard diet was fed normal food (Table 2); group 2: control group with a hypercholesterolemic diet (HD) was fed an enriched fat diet (butter 10%, cholesterol 1%, and cholic acid 0.1%); group 3: enriched fat diet and were treated with simvastatin (20 mg/Kg) for 90 days; group 4: enriched fat diet and treatment with APE (150 mg/Kg b.w./day) for 90 days; and group 5: enriched fat diet and treatment with APE (300 mg/Kg b.w./day) for 90 days. All groups were treated by oral gavage with aid.

### 2.9. Diet

The animals of the control group were fed in a standard diet (MP-77, primor, São Paulo) which consists of commercial ration for laboratory animals mice, guinea pigs and hamsters, and so forth, composed of corn meal, meat meal and soybean meal, wheat bran, sodium chloride (common salt), corn gluten meal 60, vitamin A, vitamin 12, vitamin D3, vitamin E, vitamin K3, vitamin B2, choline chloride, iron sulfate, copper sulfate, manganese sulfate, zinc oxide, calcium iodate, sodium selenite, BHT (Butylated hydroxytoluene), calcium pantothenate, niacin, and DL-methionine. The chemical composition is detailed in Table 1.

The standard diet of butter, cholesterol, and colic acid was used to induce hypercholesterolemia in animals. The percentage of butter used was 10% offering more calories to the composition of the diet, since 78% of this ingredient is lipid.

### 2.10. Sample Collection and Processing

The blood samples were collected from the retroorbital plexus of the rats, using capillary tubes 30, 60, and 90 days. After coagulation the blood was centrifuged 600 ×g for 10 minutes. The serum obtained was stored at −20°C for the determination of some biochemical markers. Serum samples were assayed for triglycerides (TG), total cholesterol (TC), high-density lipoprotein (HDL-C), aspartate aminotransferase (AST), creatinine, and urea using the Metrolab kit 23300 version 1.7 diagnostic kit analysis.

### 2.11. Histology

Histological analysis was conducted following routine methods. The tissues such as liver and kidney obtained from all the experimental groups were washed immediately with saline and then fixed in 10% buffered neutral formalin solution for 24 hrs. The organs were dehydrated with a graded series of ethanol and embedded in paraffin blocks for conventional histological processing [18]. The paraffin sections were cut at a thickness of 5 *µ*m and mounted on glass slides, stained with hematoxylin and eosin (HE). The slides were viewed under the identification of histological changes with conventional optical microscopy (Nikon YS2), and images representative of each organ were captured with a digital camera (Nikon COOLPIX L14 7.1 megapixels).

### 2.12. Determination of Malondialdehyde (MDA)

The livers of the animals were cut into small pieces and frozen at −80°C until the time of analysis. The liver lipid peroxidation in mice was determined by estimation of malondialdehyde using the thiobarbituric acid test. The liver tissue was heparinized in PBS buffer solution to 10% homogenate preparation. 250 *μ*l of the homogenate was incubated in a water bath at 37°C for 60 minutes. After incubation, 400 *μ*L of 35% perchloric acid and the samples were centrifuged at 18000 ×g for 10 minutes at 4°C. The 550 *μ*L of the supernatant was added to 200 *μ*L of 0.8% thiobarbituric acid, boiled at 95 ± 1°C for 30 minutes in a water bath and immediately cooled. Then, the measuring absorbance of the reaction mixture was at 532 nm. The standard curve was obtained using 1,1,3,3-tetra methoxy propanol. The results were expressed as nmoles of MDA per mg tissue protein [19].

### 2.13. Statistical Analysis

The data are expressed as the means ± standard error of the means (SEM). The significance of differences between animals from the groups was assessed using an analysis of variance (ANOVA), followed by the Newman-Keuls test. A value of *P* < 0.05 was considered significant.

## 3. Results and Discussion

**Figure 1:**
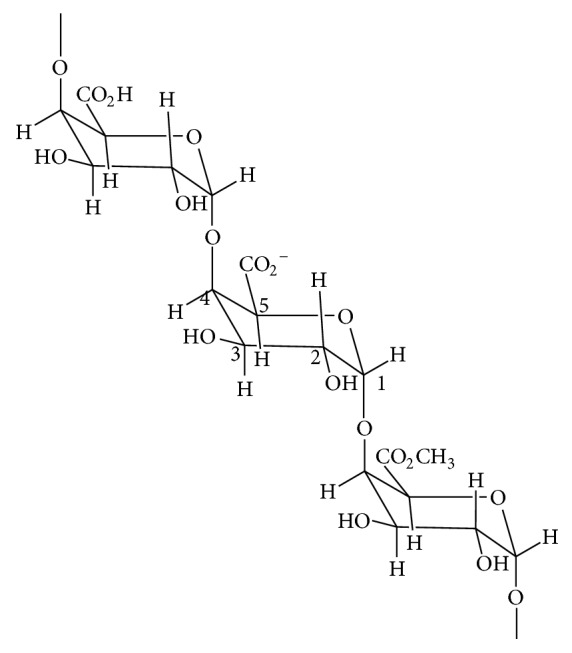
Chemical structure of pectin.

Pectin is a complex mixture of polysaccharides found in the cell walls, which produces mechanical properties due to its interaction with other cell wall components. The structure of the pectin is mainly characterized of a straight chain poly-*α*-acid (1 → 4)-D-galacturonic with various degrees of methylation of the carboxylic acid residues [20].  Figure 1 expresses the main structural representation of pectin which may have substituents. These main chain substituents may be rhamnose, arabinose, and galactose, which vary according to the sources of the pectin.

This is the first study that characterizes the pectin extracted from* Copernicia prunifera *fruits.

To determine the composition of the monosaccharides present in the pectin obtained from* C. prunifera* a sample of pectin was subjected to hydrolysis using the method of Kapoor and Joshi [17]. After neutralization and concentration, the sample was analyzed by the method of TLC, using as reference substances arabinose, galactose, and galacturonic acid. The chromatogram *R*_*f*_ values were calculated as shown in Table 2.

The chromatogram analysis confirmed the presence of galacturonic acid, galactose, and arabinose in pectin.

**Figure 2:**
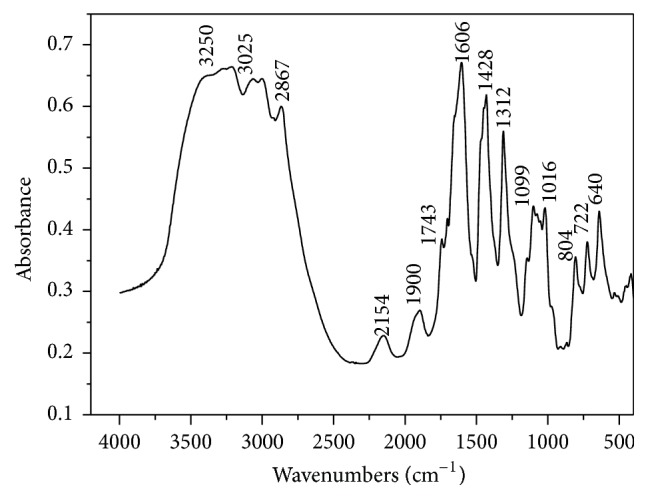
FTIR spectrum of pectin from* C. prunifera*.

An overview of the FTIR spectrum of pectin is shown in Figure 2 the “fingerprint” region of the spectrum (up to approx. 2000 cm^−1^) includes the region of 1200–1800 cm^−1^ as shown.

The IR band at approximately 1750–1350 cm^−1^ characterizes the state of carboxylic groups [21]. The band at approx. 1743 cm^−1^ is indicative of the stretching group C=O of nonionized carboxylic acid (methylated or protonated). Its ionization (formation of salt) leads to their disappearance and the appearance of stretch modes of COO^−^ in approximately 1600–1650 and 1400–1450 cm^−1^, respectively [21]. The degree of methylation (DM) is defined as the amount of ester groups compared to the total amount of acid groups and carboxylic ester and it is observed that the high intensity of the band at 1743 cm^−1^ shows that the pectin obtained is of low degree of methylation.

For confirmation of the chemical structure of the polysaccharide obtained and* C. prunifera* pectin analysis was conducted by ^1^H NMR. The spectral data are shown in Table 3.

By analyzing the ^1^H NMR spectrum of pectin and expansion, it can be confirmed that the polysaccharide obtained from unripe fruits of* Copernicia prunifera* would be a primarily polymer of poly-*α*-(1 → 4)-D-galacturonic acid. Signals around related to galacturonic acid are as follows: H-1, 49-5.2 ppm; H-4, 3.74 ppm; H-3, 3.97 ppm; H-4, 4.90 ppm; H-5 (COOMe), 4.9–5.4 ppm; H-5 (COO^−^), 4.6 ppm; and OCH_3_, 3.75 ppm [22].

**Figure 3:**
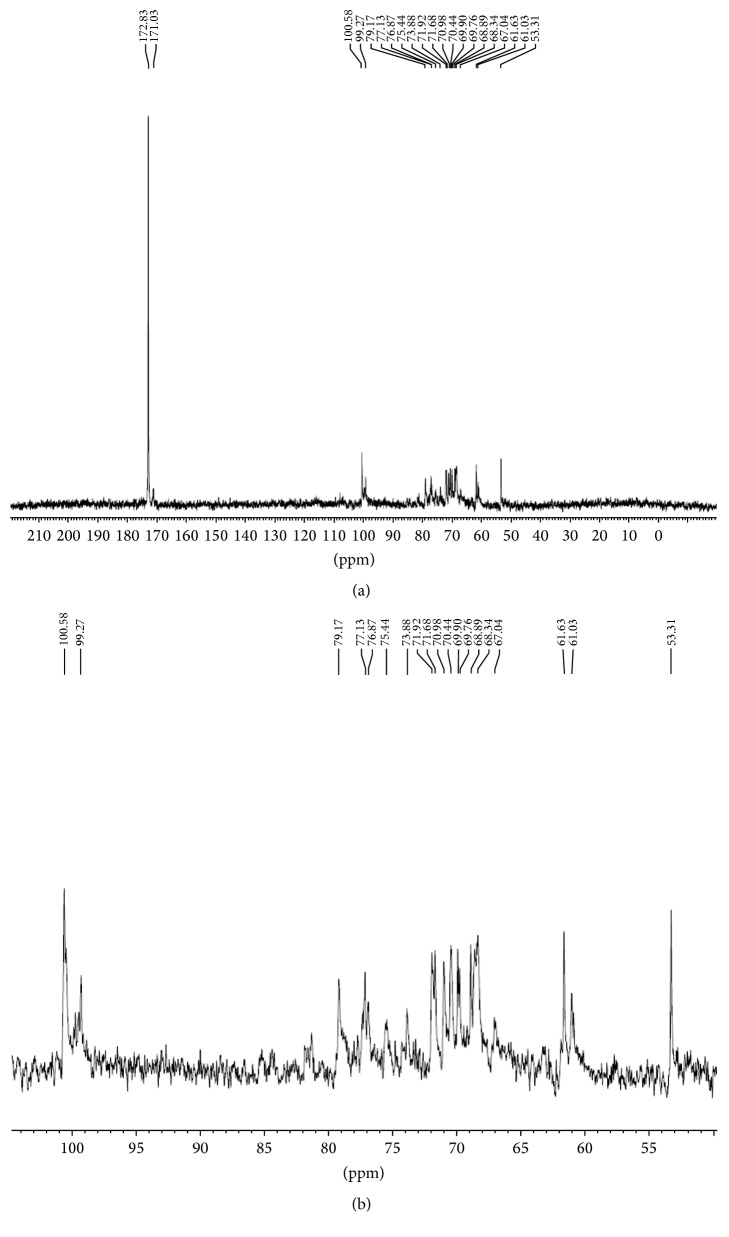
(a) ^13^CNMR spectrum of pectin obtained from* C. prunifera* fruits (D20,125 mHz). (b) Expansion of the (50–105 ppm) ^13^CNMR spectrum of pectin obtained from* C. prunifera* fruits (D20,125 mHz).

The spectrum of ^13^C nuclear magnetic resonance for the sample of pectin is shown in Figures 3(a) and 3(b). In the spectrum of the polysaccharide, a signal at about 53.3 ppm was assigned to methyl groups attached to carboxylic groups of galacturonic acid [23] and a signal at 172.8 ppm was attributed to carboxylic groups linked to methyl groups [24].

In the spectrum of the polysaccharide, major and smaller signals can be observed between the regions of about 60.0 and 110.0 ppm. The major signs are assigned to D-galacturonic acid while the smaller signs are assigned to D-galactose, as shown in Table 4 [22, 25]. These chemical shifts are in good agreement with those related to the pattern of pectin studied by Tamaki et al. [22]. There are also less intense signal assignments made by Ha et al. [25] related to arabnian but these signals are not very intense compared to the noise signals of the spectrum and therefore not all signals will be shown in the table below. There are also other signals reported by Ha et al. [25] related to other galactan carbons that are in the same situation.

**Figure 4:**
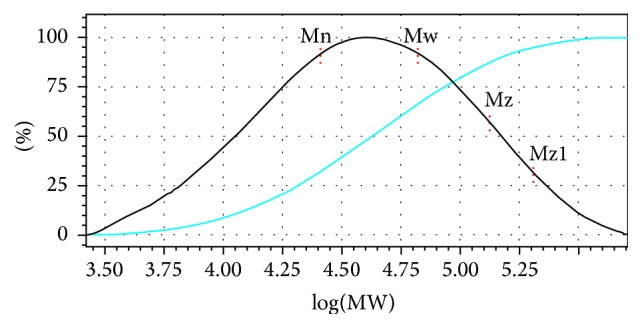
Gel permeation chromatography (GPC) of the pectin from* C. prunifera* fruits.

The molecular mass distribution of pectin was determined by GPC, whereas, for the values of the logarithms of the molar masses of pullulan standards and their molecular weight percentages, calibration curve was constructed of this analysis and the value obtained was dispersivity poly (Mw/Mn) of about 2.56 with majority peak molar mass (Mw) of 0.6 × 10^5^ g·mol^−1^ (Table 5, Figure 4).

Research in the field of natural products is being carried out in order to carry out the prospection of substances extracted from carnauba that bring benefits to human health, especially for the treatment of chronic noncommunicable diseases such as diabetes and hypercholesterolemia [16, 26].

Hyperlipidemia, whose main cause is unhealthy lifestyle, is related to an increase in the incidence of coronary, peripheral vascular disease and hepatic steatosis [27]. Thus, parameters such as high LDL-cholesterol and reduced HDL-cholesterol are shown to be relevant for the development of cardiovascular diseases [28].

More current studies demonstrate that severe hypercholesterolemia causes mutations in the genes such as the LDL receptor (LDL-R); low-density lipoprotein receptor-1 adapter protein (LDLRAP1); apolipoprotein B (apoB); and proprotein convertase subtilisin/kexin type 9 (PCSK9). It is known, however, that patients with severe hypercholesterolemia may not have mutations in these genes and may originate from polygenic, epigenetic, or acquired defects [29].

When mutations affect the LDL-R gene they interfere in the production or functioning of the LDL receptor that plays a critical role in the regulation of serum cholesterol levels by virtue of the removal of LDL from the blood. While some mutations reduce the number of synthesized LDL receptors, others impair the ability to remove LDL-R from LDL-R [30].

Similarly, mutations in the ApoB gene will trigger hereditary hypercholesterolemia or defective familial apolipoprotein B-100, while mutations in LDLRAP1 will cause autosomal recessive hereditary hypercholesterolemia. Thus, occurrence of mutations in the genes for ApoB, LDLRAP1, and PCSK9 will interfere with the synthesis or functioning of LDL receptors [30].

The onset of the formation process of atherosclerotic plaques has direct correlation with oxidative changes of lipids in LDL particles. Since the changes in these molecules are due to exposure of the lipoprotein sites to various oxidizing agents, such as superoxide anions, enzymes such as lipoxygenases, myeloperoxidase products, and hydrogen peroxides, this situation has a serious evolution when the cellular and tissue environment have diminished the antioxidant components that protect them [31].

The hypercholesterolemic diet (HD group 2) was adapted from the model proposed by Wilson et al. [32] to be an enriched diet in cholesterol-cholic acid diet which has been utilized to increase cholesterol levels in the plasma and animal experimental tissues.

In this study we chose to use the model of dyslipidemia in Swiss mice, using a hyperlipidemic diet, which has already been reported by several researchers [33–35], although it has some limitations, as it does not represent the ideal model for predisposition to atherosclerosis. However, this model provides conditions for the screening of substances that have a potential antidyslipidemic action and a lower cost and provides results with evidence that resembles those found in humans.

In such sense, this model reveals its importance as an alternative for screening low-cost hypolipidemic drugs, urging results, whose evidences resemble other correlated models in the study of dyslipidemia.

To propose a model for the screening of hypolipidemic drugs, Olivier et al. [33] used Swiss mice receiving a diet composed of butter and cholesterol to evaluate three types of classic drugs for the treatment of hyperlipidemia (fenofibrate, gemfibrozil, and nicotinic acid). The results pointed out that the effects found in Swiss mice produced similar effects in those found in humans.

In a study that sought to evaluate the efficacy of* Casearia sylvestris* leaf methanolic extract, the models used for the treatment of diet-induced dyslipidemia were C57BL/6 LDLr-null and Swiss mice. Lipidemic alterations were effective in both models causing changes in the values of TC, TG, LDL-C, and VLDL-C [34].

Changes from normal diet to hypercholesterolemic diet for 20 days caused a significant increase in TC level. All mice receiving the hypercholesterolemic diet showed significant differences in TC, TG, HDL-C, and low-density lipoprotein cholesterol (LDL-C), as they were subject to the same conditions (Table 6). The TC rate increased significantly from 106.3 mg/dL of group 1 to 210.7 mg/dL in group 2. Consequently, in the present study, the mice showed elevated serum levels of TC and also altered the LDL-C parameters. According to the study of Guedes et al. [16] there was increase in cholesterol levels of mice fed a diet containing chow enriched with 10% coconut oil (*Cocus nucifera*), 1% of cholesterol, and 0.1% cholic acid as compared to their control counterparts.

Abnormalities in lipidic values such as high LDL-C and TG and HDL-C favor the establishment of atherosclerosis which is a major promoter of diseases such as cardiovascular disease which is the leading cause of death worldwide [1, 36].

During the 30 days, daily administration of the aqueous extract of* C. Prunifera* fruits at doses of 150 and 300 mg/Kg resulted in a reduction (*P* < 0.05) of TC from 273.9 to 183.4 and 175.6 mg/dL, respectively. The same trend was observed for the period of 60 days with reduction (*P* < 0.05) from 270 mg/dL to 183.4 and 175.6 mg/dL, respectively. At 90 days, the reduction (*P* < 0.05) was only in group 4, 189.1 and 205.0 mg/dL, and group 3  220.4 mg/dL, respectively, compared to group 2 (Table 6).

Observing lipoprotein values, it can be seen that at 30 days administration of APE at doses of 150 and 300 mg/Kg in hypercholesterolemic mice showed a reduction (*P* < 0.05) in LDL-C levels from 108.1 mg/dL to 86.29 and 82.11 mg/dL, respectively. However, there was also a reduction in HDL-C levels of 84.0 and 80.0 mg/dL, respectively, both compared to group 2.

After 60 days of treatment, the reduction of TC was exclusively for lowering LDL-C (48.0 and 62.9%) and was observed only in the receiving APE groups. HDL-C was increased from 101.9 to 116.3 (14.13%) in the group receiving the dose 150 mg/Kg compared to group 1.

At the 90 days of treatment increase (*P* < 0.05) of HDL-C was perceived in all treated groups, groups 3 (96.29 mg/dL), 4 (103.7 mg/dL), and 5 (91.29 mg/dL), and reduction of LDL-C was significant only in group 3 (63.23 mg/dL) and group 4 (64.43 mg/dL) both compared to group 2.

Regarding triglycerides, the HD was not able to change this parameter. After 30 and 60 days, all treatments significantly reduced triglycerides when compared to animals fed a standard diet. However, at 90 days, only the groups which received APE (150 and 300 mg/Kg) maintained the values of triglycerides reduced compared to group 1 (Table 6).

The concentration of TC and LDL-C levels in hypercholesterolemic mice receiving APE at a dose of 150 mg/Kg had their values reduced (*P* < 0.05), as well as elevation of HDL-C compared to group 2. It was possible also to notice a reduction in TG compared to group 1.

The importance of plasma levels of cholesterol and triglycerides in the hypolipidemic effect has been demonstrated by a number of studies [13, 37].

Table 6 shows that administration of 20 mg/Kg of simvastatin in hypercholesterolemic mice significantly reduced the TC levels only 90 days later showing to be less effective than pectin. However, LDL-C levels were reduced only within 30 and 90 days.

Synthetic drugs such as statins are the most effective class of drugs for the treatment of lipid disorders, reducing the cholesterol, resulting in inhibition of HMG-CoA (3-hydroxy-3-methylglutaryl-CoA) having affinity for the active site of the liver enzyme with statins [38, 39]. Hence there is a decrease in LDL-C levels, which takes place by two mechanisms: reduced cholesterol synthesis and simultaneous increase in receptor synthesis for LDL-C in hepatic cells and increasing the clearance of LDL-C [40–43].

Therefore, research on natural products is encouraged since they indicate good therapeutic potential for global impact of diseases such as dyslipidemia [44], as the diverse biological activities and medicinal potentials of natural products are the main sources of substances which are derived from natural products quite targeted by the pharmaceutical industry [45].

The carnauba fruit contains pectin content [9], which is present in the aqueous extract used in this study, a constituent known to promote reduction of hypercholesterolemic effects [13–15]. The first proposed mechanism is that the pectin reduces serum lipids by their binding to bile acids in the intestine, consisting of cholesterol in the liver. Reducing reabsorption, as well as increased excretion of bile acids, improves the profile of cholesterolemic subjects [46]. A second possible mechanism would be to change the formation of micelles [47] contributing to the minimizing cholesterol absorption [48].

Chen et al. [49] reported the ability of polysaccharide to increase the activity of lipoprotein lipase in adipose tissue thereby reducing the amount of triglycerides in the serum of rats. Recently, Zhu et al. [15] observed the application of pectin-derived oligosaccharides (PDOs) extracted from* Crataegus pinnatifida Bunge Var.* major fruit was observed at the dose 300 mg/Kg and kunming mice fed with high fat had a significant reduction of TC and LDL-C and raising HDL-C serum in 10 weeks. Lee et al. [13] studied the pectin red ginseng in C57BL/6 obese mice fed the diet with 60% fat. The results showed a reduction in TC, LDL-C, and TG after four weeks of treatment.

In this study, as shown in Table 7, the HD induced an increase in body weight compared to group 1 (*P* < 0.05) after 90 days. An increase was not observed in consumption levels among groups. However, when examining the APE-treated group, a decrease in dietary intake was observed at a dose of 150 mg/kg which reflected the reduction in body weight of this group (Table 7).

The administration of APE (150 and 300 mg/Kg) to hypercholesterolemic mice for 90 days resulted in a significant reduction in the body weight gain in a dose-dependent manner (Table 7). The body weight increased from 32.74 g and 33.04 g at the start of the study to 43.86 g and 44.79 g at the termination in the groups administered with doses, respectively. Two test groups yielded reductions in body weight gain of 15.58% and 13.70%, respectively, as compared to the HD. The high-cholesterol diet alone yielded difference in body weight gain when compared to the normal standard diet group 1, indicating that butter and cholesterol supplementation had an appreciable effect on body weight gain. No weight reduction was observed in animals treated with simvastatin (Table 7).

The administration of APE in hypercholesterolemic animals provided a remarkable decline in body weight. Fiber consumption may play an important role in cholesterol homeostasis as an independent influencing factor. Since fibers can alter the uptake of nutrients leading to the reduction of cholesterol in the blood it is very conceivable that pectin has influenced cholesterol homeostasis by a simple but efficient method for concealing food intake and additionally change in body weight.

A previous study by Shehata and Soltan [50] reported that mice fed a diet enriched with 1% cholesterol, 16% fat, and 0.2% colic showed significant body weight gain when supplemented in the diet with purslane and celery (fresh and seeds). These results can be explained by the presence of fibers that slow the intestinal transit velocity favoring satiety and lower weight gain.

The administration of APE at doses of 150 and 300 mg/Kg to hypercholesterolemic mice caused a significant decrease in body weight gain (43.86 and 44.79 g) as compared with group 2 (51.96 g). This result suggests that the reduced weight gain may be due to presence of pectin in carnauba fruits [9], delaying the absorption of some nutrients such as lipids [51].

Our results are in agreement with Fidele et al. [52] that used the aqueous extract of fresh leaves of* F. glumosa* (Moraceae), which, in addition to its hypolipidemic effects, showed a reduction of 15.51% by weight after 4 weeks of treatment at a dose of 300 mg/Kg.

Lee et al. [13] administered lyase-modified pectin extracted from red ginseng in obese mice using a high fat diet. After 4 weeks the reduction of weights was significantly lower compared to the group that received only high fat diet. Feed intake did not differ significantly between groups, whereas our experimental study showed difference in intake only in group 4 in the 90 days.

However, there is a significant number of findings to increase consumption of dietary fiber with protective potential to avoid the development of hyperphagia and obesity in mice and rats, whose diet is the basis of high fat diets [37, 53, 54]. Our study endorses the efficacy of dietary fiber pectin with regard to weight reduction in mice originally using a hypercholesterolemic and obesogenic diet.

In our work some of liver toxicity parameters (enzyme AST) and renal metabolites (urea and creatinine) are analyzed, as well as histopathological examination of the liver and kidneys of the animals studied.

In study of preclinical toxicity, the appearances of abnormalities in serum liver enzyme activities are considered sensitive indicators of hepatobiliary changes [55]. The liver is the main organ responsible for the maintenance of cholesterol homeostasis, and thus the appearance of liver enzymes in the bloodstream is a strong indicator that the cell membranes were broken with the release of these enzymes [55].

Usually the AST and ALT hepatic enzymes increase as a result of the increase in dietary cholesterol content [56]. Increased serum extravasation, especially AST, may also indicate damage to skeletal myofibrils [57].

However, data in Table 8 illustrated that liver function of hypercholestermic mice fed with hypercholesterolemic diet did not change the blood levels of AST. Significant change was observed in the group receiving simvastatin with elevation of 85.57 U/L for 190.7 U/L of these parameters compared to group 2 indicating a potential risk of liver injury by standard medication. There was no elevation of these enzymes in the receiving APE groups.

The hypercholesterolemic diet significantly increased serum urea to 55.86 mg/dL. However, only the animals that received APE (150 and 300 mg/Kg) had their decreased concentration of this parameter (46.43 and 37.43 mg/dL) which is comparable with group 2 (47.14 mg/dL). The diet significantly reduced serum creatinine levels (0.62 mg/dL) compared to group 1 (0.88 mg/dL), as well as treatments with simvastatin (0.58 mg/dL) and APE dose (150 mg/Kg) (0.55 mg/dL). However, administration of the highest APE dose (300 mg/Kg) promoted regularization of this indicator with 0.84 mg/dL, avoiding the progression of renal dysfunction (Table 8).

Hypercholesterolemia has been involved in the damage to the kidney functions [58]. This is evident in Table 8 and the hypercholesterolemia induced by HD diet of mice led to kidney dysfunction measured by the alteration in the concentrations of urea and creatinine in the blood. Urea is a by-product from protein breakdown and when associated with elevated creatinine levels may indicate muscle impairment.

Urinary parameters that are the basis of urea are considered quite useful, considering that such substance has its resorption in the proximal portion of the contoured tubule and stands out among the most important solutes that are concentrated in the renal medulla, a region that is especially sensitive to hypoxia [59].

Since a higher than normal level of blood urea and creatinine will demonstrate kidney damage, the most frequently utilized clinical indices for assessing renal capacity rely on concentration of urea in the serum.

The addition of dietary fiber for the treatment of hypercholesterolemia is expected to be effective for the recovery of renal function by the improvement of lipid metabolism. Samout et al. [60] reported that in rats treated with pectin under high fat diet administration all these biomarkers were restored to near normal values.

The administration of dietary fiber favors the reduction of blood urea levels due to the increase of excretion of nitrogen through feces or through the renal exception [61]. This mechanism can be explained by the probable presence of colonic fermentation of the fibers leading to the growth of the population of intestinal bacteria that require nitrogen for the protein synthesis, retaining this component in the lumen and increasing its excretion [62, 63].

In a study using pectin extracted from citrus fruit that evaluated the therapeutic effect in the reduction of renal dysfunction, oxidative stress was caused by octylphenol administration, and it was found that the polysaccharide has antioxidant activity and minimizes the toxic effects induced by octylphenol [64].

Thus, it can be suggested that pectin present in the fruits of carnauba has the potential to improve excretion of urea, reflecting the preservation of the integrity of renal function when subjected to a hypercholesterolemic diet.

Previous studies reliably showed the antioxidant potential of oligosaccharides [65] and polysaccharides [66], in food. Consequently, taking into account the relative increase of reactive oxygen species with dyslipidemia [67], in this way the levels of lipid peroxidation were assessed by malondialdehyde (MDA) reaction with thiobarbituric acid by TBARS assay in homogenates of liver of hypercholesterolemic animals receiving the extract from carnauba fruits.

The hypercholesterolemic diet significantly increased MDA levels by 0.39 nmol/g compared to group 1. The results showed that lipid peroxidation of both doses (150 and 300 mg/Kg) used reduced malondialdehyde concentration of liver tissue (0.21 and 0.22 nmol/g) when compared with HD (0.39 nmol/g) indicating a protective effect against oxidative stress caused by hypercholesterolemic diet (Table 8). This result is relevant, since malonaldehyde (MDA), a product of lipid peroxidation, is extremely reactive possibly causing structural alterations of the cellular components by reaction with the amino group of proteins, phospholipids, and nucleic acids [68, 69].

A high fat diet may increase plasma lipids causing oxidative stress prompting an imbalance in the production of oxidizing compounds often not accompanied by this removal capacity. Consequently, there is the oxidative cell damage and tissue due to the oxidation of biomolecules with indirect loss of their biological imbalance functions. This process is one of the main outcome results of lipid peroxidation and it can often leads to increased production of oxygen free radicals [70].

Thus, it can be considered that the protective effect against oxidative stress is due to the reduction of blood lipid levels, since hypercholesterolemia leads to increased production of reactive oxygen species, due to the action of enzymes such as NADPH oxidase, xanthine oxidase, and mitochondrial enzymes from reactive oxygen species producing, for example, anion superoxide [71].

**Figure 5:**
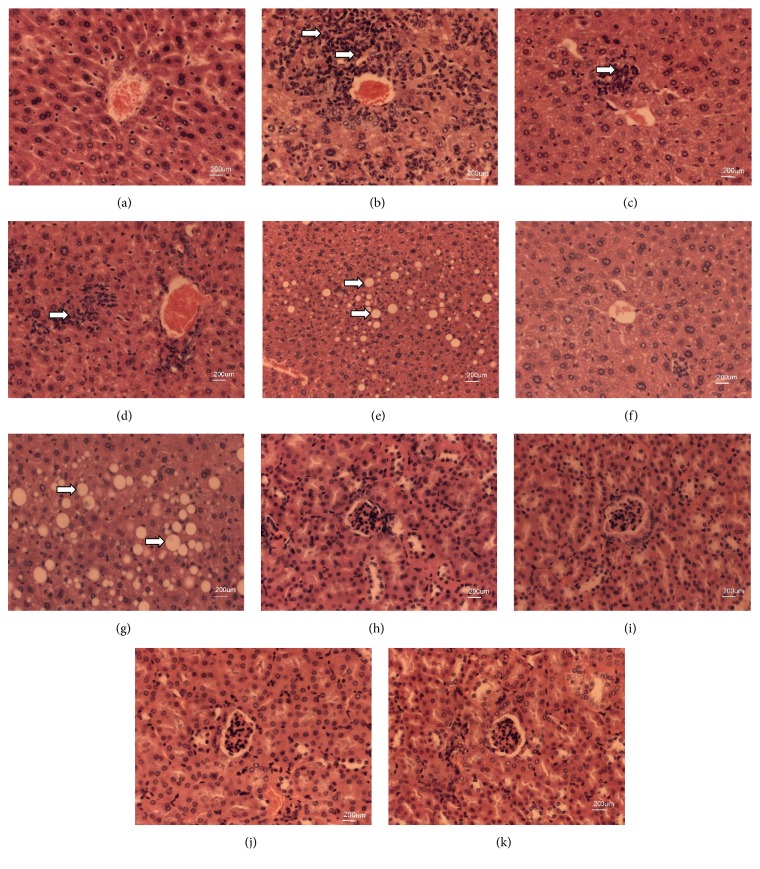
Histopathology images of liver and kidney hypercholesterolemic animals treated with APE. ((a)–(d)) Liver. (a) Standard diet, (b) hypercholesterolemic diet, (c) simvastatin, and (d) APE, aqueous fruit pulp extracts. Representative areas of inflammatory process consisting of neutrophils, macrophages, lymphocytes, and plasma cells (white arrows). ((e)–(g)) Liver. (e) Hypercholesterolemic diet, (f) simvastatin, and (g) APE, aqueous fruit pulp extracts. HE, fat droplets representing hepatic steatosis. ((h)–(k)) Kidney. (e) Standard diet, (f) hypercholesterolemic diet, (g) simvastatin, and (h) APE, aqueous fruit pulp extracts. HE, 200x.

Figure 5 shows the photomicrographs of the livers and kidneys of hypercholesterolemic animals treated with APE from* C. prunifera*. Histopathological analysis revealed that the kidneys of all animals used, showed no cytotoxic or architectural changes (Figures 5(h), 5(i), 5(j), and 5(k)).

Inflammation of hepatic tissue caused by the hypercholesterolemic diet was lower in the groups receiving APE (Figure 5(d)) when compared to the group fed with HD (Figure 5(b)), and also the number of affected animals was lower in the group with the highest dose (APE 300 mg/Kg). This may suggest an anti-inflammatory effect of the pectin present in APE (Figure 5(d)).

According to the histopathological analysis, administration of the hypercholesterolemic diet caused extensive accumulation of lipid droplets and APE treatment resulted in a lower number of animals affected with steatosis (Figure 5(g)) when compared to the HD group (Figure 5(e)), although this did not vary in intensity (discrete).

As found by Zhu et al. [72], pectin and pectin hydrolysate treatment improved hepatic steatosis. However, the effects of the treatments were not significant. The formation of fatty deposits in the liver is directly related to the events of lipid peroxidation [73] and cardiovascular diseases [74].

The liver of control diet showed no cytotoxic or architectural changes (Figure 5(a)). With respect to the liver of the animals that received only hypercholesterolemic diet, the most significant lesion was observed and subacute inflammatory process varied from mild to severe. It is likely that trigger inflammatory processes have correlation with lipotoxicity (injury or cell death, having as cause the free fatty acids as well as their metabolites) [75]. These free fatty acids can be brought, as suggested by Yamaguchi et al. [76], by an enzyme inhibition mechanism diacylglycerol acyltransferase 2, related to the biosynthesis of lipids, which would lead to a reduction in murine models of hepatic steatosis but concomitantly lead to increased levels of free fatty acids with toxic potential to cause liver inflammation [77].

Although the histology results in liver of mice treated with APE indicates that the treatments were not able to significantly alter the inflammatory process compared to the standard diet, nevertheless, the possibility can be suggested that gave a better response against the aggression when compared to animals fed with HD. Furthermore, it was observed lowest intensity of inflammation in the group receiving the higher dose.

It is known that inflammatory processes in the liver can have several causes; however, the most relevant factor is related to the development of inflammation comprising oxidative stress [78], as observed in HD group when analyzing the results of lipid peroxidation. The elevation of malonaldehyde contributes to the activation of NF-kB, which is responsible for regulating the expression of various cytokine with proinflammatory actions, such as TNF-*α* and interleukin-8 (IL-8) [79].

It is also known that the hepatic vulnerability resulting from secondary injuries from the effects of cytokines or from oxidative stress is more pronounced due to the accumulation of triglycerides in the hepatocytes, as occurs in hepatic steatosis [8].

Therefore, this result indicates that the APE has potential for reduction in total cholesterol and triglyceride levels and oxidative stress shows a potential liver protective effect, since they reduced deleterious activity in the liver tissue according to histological analysis by light microscopy, and the results obtained by determination of AST enzyme are useful in the detection of hepatocellular inflammation.

In addition, the contribution of this pectin compared to previous pectin is due to the fact that, despite having low molecular weight and low degree of esterification, the pectin contained in the fruits of carnauba proved very promising in lowering serum lipid. Considering this point of view the pectin presence of higher molecular weight and higher degree of esterification shows better performances.

We attributed the hypocholesterolemic effects of this study to pectin that has a relevant role in the excretion of bile acids and reduction of their intestinal reabsorption, increase in the viscosity of the digestion content, reducing the absorption of fat with consequent reduction of body weight, and increased sensation of satiety and possibly altering the activity of enzymes that are fundamental to digestion.

Thus, this study sought to collaborate with preclinical investigations of this species and supports the potential use of pectin of fruits of* C. prunifera* as a candidate for phytotherapy for the treatment of hyperlipidemias.

## 4. Conclusions

The pectin content obtained from the pulp of unripe fruits of* C*.* prunifera* of this study showed a value of 2.9%. For the analysis of absorption spectra by FTIR, it was observed that pectin has several carbonyl groups in the form of carboxylate and esterified. The thin layer chromatography carried out in hydrolysates of pectin of the study presented in *R*_*f*_ the galacturonic acid patterns, galactose, and arabinose. Such substances are monomers constituting the polysaccharide chain.

IR spectroscopy and ^1^HNMR were effective in characterizing the pectin sample. DM for the sample by the IR spectroscopy was characterized the low-methoxyl pectin. By ^13^C NMR spectroscopy, different groups of polymers now were identified in the chains of pectic polysaccharides obtained. The molecular peak was determined by GPC to the value of 0.6 × 10^5^ g·mol^−1^.

The in vivo study results show that APE from the* C*.* prunifera* showed promising results in the treatment of diseases associated with lipid metabolism disorders and possibly ant atherosclerotic. At 90 days APE was able to reduce total cholesterol levels, LDL-C and triglycerides in serum, and MDA and inflammation in the liver tissue as well as body weight in hypercholesterolemic animals.

This is the first in vivo study with APE of* C*.* prunifera* suggesting its potential as an alternative therapeutic agent for hypercholesterolemia. The doses used of APE (150 and 300 mg/Kg b.w./day) showed no renal toxicity and liver toxicity to animals. However, other preclinical and clinical studies are necessary to ensure and validate therapeutic applicability of this substance, as well as in vivo testing of the isolated pectin.

## Figures and Tables

**Table 1:** Composition of the standard diet.

Standard diet	Composition (%)
Moisture	13
Crude protein	23
Ethereal extract	3
Fibrous matter	10
Carbohydrate	43.5
Mineral matter	7.5
Total	100

*Source*. Primo MP 77 feed ration label, São Paulo.

**Table 2:** *R*_*f*_ values of hydrolysed pectin extracted from *C. prunifera* fruits.

Sample	*R*_*f*_
Galacturonic acid	0.32
Galactose	0.40
Arabinose	0.48

**Table 3:** ^1^H resonance of pectin in the fruits of *C. prunifera *characterization of pectin by ^1^H NMR.

Chemical shifts (in ppm)	Proton residue of galacturonic acid
4.9–5.2	H-1
3.74	H-4
3.97	H-3
4.90	H-4
4.9–5.4	H-5 (COOMe)
4.60	H-5 (COO^−^)
3.75	OCH_3_

**Table 4:** ^13^CNMR spectrum for sample of pectin from *C. prunifera*.

Polymer	Carbon	Chemical Shifts (in ppm)
Galacturonan	C-6 free	172.8
Galacturonan	C-6 ester	171.0
Arabinan	C-1	—
Galacturonan	C-1	100.5
Arabinan	C-4	84.5
Arabinan	C-4	83.0
Arabinan	C-2	81.0
Galacturonan	C-4	79.1
Galactan	C-4	77.1
Arabinan	C-3	76.8
Galacturonan	C-3	71.6
Galacturonan	C-5	73.8
Galacturonan	C-2	68.3
Arabinan	C-5	67.0
Galactan	C-6	61.6
Arabinan	C-5	61.0
Galacturonan	OCH_3_	53.5

“—”: not detected.

**Table 5:** Mean values of the molecular weight of the pectin *C. prunifera*.

Total weight	Mn	Mw	Mz	Mz1	Mw/Mn
	25721	65924	133266	203367	2.56310

Number average molecular mass (Mn); weight average molecular mass (Mw); molecular weight *Z* average (Mz); molecular weight *Z* + 1 average (Mz1).

**Table 6:** Effects of APE/simvastatin treatment on serum total cholesterol and triglyceride levels in mice fed with HD for 30, 60, and 90 days.

Parameters (days)	SD	HD	SIMV	APE150	APE300
Cholesterol level					
Baseline	106.3 ± 3.75	210.7 ± 6.35^a^	199.0 ± 5.08^a^	197.6 ± 5.44^a^	201.9 ± 6.29^a^
30 days	167.7 ± 6.02	273.9 ± 9.12^a^	197.0 ± 3.05^a,b^	183.4 ± 4.29^b^	175.6 ± 4.74^b^
60 days	155.9 ± 7.49	270.0 ± 11.98^a^	252.9 ± 5.58^a^	183.3 ± 7.88^b^	185.3 ± 10.95^b^
90 days	175.0 ± 8.93	220.4 ± 8.49^a^	191.7 ± 8.67^b^	189.1 ± 5.14^b^	205.0 ± 5.55^a^
Triglycerides level					
Baseline	102.0 ± 9,93	79.14 ± 3,01	107.1 ± 3,95	80.86 ± 9,86	84.00 ± 5,81
30 days	157.6 ± 9.18	147.3 ± 8.27	89.14 ± 2.34^a,b^	65.71 ± 7.63^a,b^	67.29 ± 4.32^a,b^
60 days	180.6 ± 8.53	171.5 ± 14.41	127.9 ± 7.02^a,b^	95.00 ± 7.11^a,b^	103.7 ± 9.31^a,b^
90 days	192.0 ± 14.44	182.1 ± 19.81	161.0 ± 19.24	105.0 ± 7.72^a,b^	101.9 ± 7.14^a,b^
HDL level					
Baseline	74.57 ± 4.09	83.86 ± 3.51	83.43 ± 2.27	85.86 ± 3.72	87.29 ± 1.66
30 days	93.71 ± 2.50	108.1 ± 3.88^a^	97.86 ± 4.28^b^	84.00 ± 3.02^a,b^	80.00 ± 2.31^a,b^
60 days	90.71 ± 3.54	101.9 ± 5.00	115.1 ± 5.44^a^	116.3 ± 6.00^a^	101.6 ± 3.55
90 days	100.6 ± 4.38	70.57 ± 2.43^a^	96.29 ± 3.06^b^	103.7 ± 3.15^b^	91.29 ± 1.69^b^
LDL level					
Baseline	14.10 ± 0.81	111.0 ± 7.25^a^	94.14 ± 4.98^a^	95.54 ± 4.15^a^	97.77 ± 5.96^a^
30 days	42.49 ± 7.01	136.3 ± 12.61^a^	81.31 ± 3.67^a,b^	86.29 ± 6.06^a,b^	82.11 ± 5.26^a,b^
60 days	34.65 ± 3.27	133.8 ± 9.35^a^	112.1 ± 9.95^a^	48.00 ± 8.68^b^	62.97 ± 9.17^b^
90 days	36.03 ± 5.39	113.4 ± 8.41^a^	63.23 ± 6.29^a,b^	64.43 ± 8.14^a,b^	93.34 ± 6.08^a^

SD, standard diet; HD, hypercholesterolaemic diet; SIMV, simvastatin (20 mg/Kg/day, i.g.); TC, total cholesterol; TG, triglycerides. APE 150, APE 300 (aqueous fruit pulp extracts 150 and 300 mg/Kg/day, i.g.). Values are given as the mean ± SEM of 7 mice per group. To analyze the significance of the differences between the samples of authors used analysis of variance (ANOVA) followed by the Newman-Keuls comparison test, ^a^*P* < 0.05 versus the SD group; ^b^*P* < 0.05 versus HD group.

**Table 7:** Effects of APE on weight and total weight gain of mice submitted to the hypercholesterolemic diet.

Groups	Initial weight (g)	Final weight (g)	Total weight gain (g)	Food intake (g/mice/day)		
				30 days	60 days	90 days
SD	30.89 ± 1.74	42.34 ± 2.25	11.46 ± 2.68	5.39 ± 0.21	5.52 ± 0.17	5.34 ± 0.07
HD	32.8 ± 0.56	51.96 ± 1.8^a^	19.16 ± 3.69	5.22 ± 0.22	5.38 ± 0.25	5.46 ± 0.07
SIMV	31.41 ± 0.59	48.3 ± 2.15	16.89 ± 4.29	5.53 ± 0.22	5.65 ± 0.20	5.66 ± 0.09
APE 150	32.74 ± 0.54	43.86 ± 0.53^b^	11.11 ± 1.98	4.73 ± 0.22	5.0 ± 0.06	4.62 ± 0.17^a,b^
APE 300	33.04 ± 0.83	44.79 ± 0.81^b^	11.74 ± 2.64	4.93 ± 0.26	5.77 ± 0.20	5.67 ± 0.16

SD, standard diet; HD, hypercholesterolaemic diet; SIMV, simvastatin (20 mg/Kg/day, i.g.); APE 150, APE 300 (aqueous fruit pulp extracts 150 and 300 mg/Kg/day, i.g.). Values are given as the mean ± SEM of 7 mice per group. To analyze the significance of the differences between the samples of authors used analysis of variance (ANOVA) followed by the Newman-Keuls comparison test, ^a^*P* < 0.05 versus the SD group; ^b^*P* < 0.05 versus HD group.

**Table 8:** Effects of APE on the relative liver weight, malondialdehyde in liver tissue and serum renal metabolites of mice submitted to the hypercholesterolemic diet.

Parameters	SD	HD	SIMV	APE150	APE300
RLW	3.76 ± 0.15	4.88 ± 0.35^a^	4.17 ± 0.18	4.29 ± 0.16	4.36 ± 0.11
MDA	0.24 ± 0.02	0.39 ± 0.05^a^	—	0.21 ± 0.01^b^	0.22 ± 0.01^b^
AST	95.57 ± 9.90	85.57 ± 12.24	190.7 ± 14.85^a,b^	95.00 ± 11.98	105.4 ± 12.46
UREA	47.14 ± 1.46	55.86 ± 1.22^a^	53.57 ± 1.34^a^	46.43 ± 2.69^b^	37.43 ± 1.90^a,b^
CREAT	0.88 ± 0.04	0.62 ± 0.06^a^	0.58 ± 0.07^a^	0.55 ± 0.04^a^	0.84 ± 0.05^b^

SD, standard diet; HD, hypercholesterolaemic diet; SIMV, simvastatin (20 mg/Kg/day, i.g.); RLW, relative liver weight (relative liver weight = liver weight in percent of body weight); MDA, malondialdehyde; AST, aspartate aminotransferase; CREAT, creatinine. APE 150, APE 300 (aqueous fruit pulp extracts 150 and 300 mg/Kg/day, i.g.). Values are given as the mean ± SEM of 7 mice per group. To analyze the significance of the differences between the samples of authors used analysis of variance (ANOVA) followed by the Newman-Keuls comparison test, ^a^*P* < 0.05 versus the SD group; ^b^*P* < 0.05 versus HD group.
