# Corrigendum to “Antihypercholesterolemic Effects of Fruit Aqueous Extract of *Copernicia prunifera* (Miller) H. E. Moore in Mice Diet-Induced Hypercholesterolemia”

**DOI:** 10.1155/2017/2486328

**Published:** 2017-10-11

**Authors:** Raquel Teixeira Terceiro Paim, Stephen Rathinaraj Benjamin, Davide Rondina, Márcia Maria Mendes Marques, Daniel de Araújo Viana, Maria Leônia da Costa Gonzaga, Ícaro Gusmão Pinto Vieira, Francisca Noélia Pereira Mendes, Paula Alves Salmito Rodrigues, Maria Izabel Florindo Guedes

**Affiliations:** ^1^Northeast Biotechnology Network, Graduate Program of Biotechnology, State University of Ceará, Itaperi Campus, 60714-903 Fortaleza, CE, Brazil; ^2^Laboratory of Biotechnology and Molecular Biology and Health Science Center, State University of Ceará, Itaperi Campus, 60714-903 Fortaleza, CE, Brazil; ^3^Faculty of Veterinary, State University of Ceará, Itaperi Campus, 60714-903 Fortaleza, CE, Brazil; ^4^Federal University of Piauí, Campus Senador Helvídio Nunes de Barros, Junco, 64607-670 Picos, PI, Brazil; ^5^Laboratory of Veterinary Pathology, State University of Ceará, Itaperi Campus, 60740-000 Fortaleza, CE, Brazil; ^6^Federal University of Ceará, University Campus of Pici, 60356-000 Fortaleza, CE, Brazil; ^7^Laboratory of Natural Products, State University of Ceará, Itaperi Campus, 60740-000 Fortaleza, CE, Brazil

In the article titled “Antihypercholesterolemic Effects of Fruit Aqueous Extract of* Copernicia prunifera* (Miller) H. E. Moore in Mice Diet-Induced Hypercholesterolemia” [1], Dr. Paula Alves Salmito Rodrigues was missing from the authors' list. The corrected authors' list is shown above and updated in place.
